# Use of digital platforms and social media as a source of information on children's oral health by parents: a cross-sectional survey analysis in a Spanish sample

**DOI:** 10.3389/froh.2026.1754009

**Published:** 2026-03-24

**Authors:** Anabella Reyes Ortiz, María Fe Riolobos González, Lourdes García Navas Fernández de la Puebla, Andrea Álvarez Alonso, Fátima Cruz-Cruz, Andrea Martín-Vacas

**Affiliations:** 1 Master Programme in Paediatric Dentistry, Universidad Alfonso X El Sabio, Madrid, Spain; 2 Facultad de Odontología, Universidad Alfonso X El Sabio, Villanueva de la Cañada, Spain

**Keywords:** digital platforms, oral health, parenting, pediatric dentistry, social media

## Abstract

**Aim:**

To identify the primary social media and digital platforms used by parents and/or caregivers of children, analysing search habits regarding interests in OH, the frequency of usage, and the level of reliability attributed to the consulted platforms.

**Methods:**

A cross-sectional analysis with an anonymous survey was conducted. Following a non-probabilistic sampling, parents or caregivers were invited to complete a 14 questions’ survey. A descriptive and analytical statistical analysis were conducted with a 95% level of confidence.

**Results:**

A total of 112 surveys were obtained, mainly filled by females (70.5%) between 31 and 40 years (57.1%). Mostly of the respondents (61.3%) stated that the search for information about their children’s OH, being the main reason for searching interest in the topic, and the device more used the mobile phone. Maternity websites were the first search choice, followed by Instagram® and scientific databases. Half of the studied sample (52.7%) consider the information as not very reliable or not reliable at all, most respondents stated that they consulted with family and friends.

**Conclusion:**

Most parents search for information about their children’s OH on online platforms. Only 21.74% of respondents verified the information with a paediatric dentist, rating the information as not very reliable or moderately reliable.

## 1 Introduction

Oral health (OH), as defined by the World Health Organization (WHO), is an essential component of overall health that enables individuals to perform vital functions—including eating, breathing, and speaking—while also exerting a significant influence on psychosocial well-being, self-esteem, and social interaction. This dimension of health evolves across the lifespan, forming an inseparable part of general health and shaping individuals’ capacity to engage fully in society and reach their maximum potential (1). More recently, the World Dental Federation (FDI) has updated its definition of OH as a multifaceted construct encompassing the ability to speak, smile, smell, taste, touch, chew, and swallow, as well as to express a wide range of emotions with confidence and without pain, discomfort, or disease affecting the craniofacial complex (2). According to FDI, the physiological interplay between OH and sensory modalities, such as tactile mechanoreception and olfactory acuity, is a critical determinant of an individual’s psychosocial well-being and nutritional autonomy. Maintaining oral homeostasis is essential for the preservation of craniofacial sensory functions, including the ability to perceive textures and aromas, which are fundamental to the overall quality of life.

Health information–seeking behaviour among parents is shaped by perceived needs, previous experiences, literacy level, and accessibility to trusted sources. During pregnancy and early parenthood, many mothers report heightened uncertainty and anxiety, which often translates into an increased search for guidance on childcare and health-related topics (3–5). Prior research shows that parental concerns vary across settings, for example, Israeli parents prioritize educational issues, whereas French parents focus more on cognitive and developmental aspects (6). Studies conducted in Ireland (7), the United Kingdom (8), and Australia (9) similarly indicate that parents frequently turn to online sources to obtain information about child health, including OH. Additionally, it has been demonstrated that first-time expectant mothers are more likely to turn to social media as a source of information on childcare and children's health (10).

Social media and digital platforms have become integral to many aspects of daily life, influencing numerous decisions made. In terms of healthcare, the advantages of social media include enhanced connectivity, increased community support, the formation of international working groups, and the ability to engage an unlimited number of participants. Moreover, their cost-effectiveness and immediate accessibility make these platforms a convenient and practical communication tool. However, certain limitations exist, such as concerns over confidentiality, invasion of privacy, low accuracy, and the need for rigorous quality control of the information (11). Among the most widely used social media platforms, Facebook®, Instagram®, and YouTube® stand out. Facebook®, with 1.22 to 2.9 billion active users (12–15). Social media platforms enable users to easily share photos and videos, temporary stories and hashtags. Digital platforms and social media have become major channels through which parents access health information. Their immediacy, accessibility, and perceived peer support make them particularly appealing for young adults, especially women under 34 years, who are among the most active users (16). However, the reliability of the information available online is highly variable. Although these platforms allow rapid dissemination of content, they also present risks related to limited accuracy, potential misinterpretation, commercial bias, and lack of professional oversight (14, 15, 17). Moreover, children and adolescents increasingly access the Internet and social networks at an early age. A recent study showed that among English children aged 12–15 years, the Internet (43.4%) and social networks (14.3%) were significant sources of OH information after parents (18), further amplifying the impact of digital environments on OH knowledge.

Evidence regarding OH promotion through social media is growing, though heterogeneous. Some studies highlight the potential of digital communities to facilitate health education, peer support and engagement, whereas others underscore concerns related to misinformation and the paucity of quality control mechanisms (17). Research evaluating maternal and child health applications, for example, has demonstrated substantial variability in functionality, content quality, and security, with none fully meeting established standards (19). However, despite the increasing integration of digital tools into everyday life, there is limited evidence from the Spanish population, particularly regarding how parents of children use social media and digital platforms to seek OH-related information. Given the high prevalence of early childhood oral diseases and the growing dependence on digital sources for health information, understanding parents’ information-seeking patterns is crucial. Exploring these behaviours can provide insights for dental professionals, inform the development of evidencebased digital resources, and guide strategies to counter misinformation and promote reliable OH guidance. The aim of this study was to identify the primary social media and digital platforms used by parents and/or caregivers of children, analysing search habits regarding interests in OH, the frequency of usage, and the level of reliability attributed to the consulted platforms.

## 2 Material and methods

### 2.1 Study design and ethical considerations

A cross-sectional analytical study has been conducted using an online questionnaire. The study protocol was approved by the University of Alfonso X El Sabio (UAX) Research Ethics Committee (Resolution 2024_4/266), and the manuscript has been prepared according to the consensus-based checklist for reporting of survey studies (CROSS) (20) and STROBE statements (21) (Supplementary Appendix 1). The research was conducted at the Clinic of Dental Specialties of Alfonso X El Sabio University (UAX), within the facilities of the Paediatric Dentistry Master’s programme.

### 2.2 Study population and sample size calculation

The required sample size was calculated with G*Power software (version 3.1.9.7.), with *a priori* power analysis, Chi-square (ꭓ^2^) tests family, with a ratio 1.5, 5% alpha error and 80% power, obtaining a required sample size of 93 subjects. In order to prevent lost subjects or data, it was decided to increase the sample size by 10%, requiring 103 subjects. A non-probabilistic consecutive case sampling strategy was used. All parents and/or legal guardians who attended the Paediatric Dentistry Master’s Clinic at Universidad Alfonso X el Sabio during routine check-ups or treatments over the 5-month recruitment period were invited to participate in the order in which they presented to the clinic. Occasional caregivers and child companions who were not parents or legal guardians were excluded. Parents or caregivers with language barriers or intellectual difficulties in completing the questionnaire were also excluded. Written consent was incorporated into the digital questionnaire for parents or legal guardians. Non-probabilistic consecutive sampling approach was selected due to the pragmatic nature of clinical recruitment. Mothers are typically the primary caregivers attending paediatric dental visits, which explains the higher proportion of female respondents. Similar gender distributions have been reported in previous research on parental digital health-seeking behaviours. This gender imbalance is consistent with previous research showing that mothers are the primary users of digital platforms for seeking child-health information, as documented both in the scoping review by Frey et al. (22) and in the earlier findings of Bidmon and Terlutter (23), who similarly reported higher engagement of women in online health-information seeking.

### 2.3 Questionnaire description

An anonymous survey was conducted including 14 questions (the questionnaire in English and original Spanish can be consulted in Supplementary Appendix 2), directed at parents and/or legal guardians of children. The first four questions gathered demographic information about the respondents, including gender, age, educational level, and monthly family income. The next two questions addressed their daily use of social media and digital platforms. The remaining eight questions linked social media use to online searches concerning general and OH topics. Some questions allowed for multiple-choice responses. The questionnaire consisted exclusively of close-ended questions. All 14 items were formatted as multiple-choice or single-answer options to facilitate completion, ensure standardization of responses, and allow for quantitative analysis. No open-ended questions were included.

The questionnaire used in this study was specifically developed by the research team based on existing knowledge and literature (15) regarding parental information seeking behaviours, digital health use, and maternal electronic practices, including recent evidence on parents’ social-media use for child health (platforms, reasons for searching, verification practices, and perceived reliability) synthesized by Frey et al. (22) and population-based data describing platform uptake and daily engagement among parents and women of childbearing age reported by Waring et al. (24). The questionnaire was developed *ad hoc* by the research team, following a multi-step validation process to ensure methodological rigor. Content validity was assessed by a panel of three paediatric dentistry specialists with clinical and academic experience. Each expert independently evaluated the clarity, relevance, representativeness, and appropriateness of the items using a structured qualitative review. Their recommendations led to refinement and consolidation of several questions to ensure conceptual accuracy and eliminate redundancy. Face validity was assessed through a pilot test involving 12 parents who met the inclusion criteria and represented the target population. Participants completed the questionnaire using the same Google Forms® interface employed in the study and were asked to report any difficulties related to comprehension, wording, flow, and usability. Overall, the questionnaire was perceived as clear and easy to complete, and only minor wording adjustments and formatting refinements were required. This process ensured that the final instrument was understandable, user-friendly, and appropriate for the intended population. This multi-step approach is also consistent with international recommendations for evaluating content validity and comprehensibility, such as the COSMIN methodology (25), which emphasises expert judgement and cognitive/pilot testing as the key quality criteria when instruments do not measure latent constructs. Although COSMIN is primarily intended for PROMs, its guidance on relevance, comprehensiveness and clarity supports the methodological steps undertaken in the present study. Because the instrument consisted exclusively of descriptive, categorical, non-latent variables (e.g., type of platform used, frequency, verification method), the questionnaire was not designed to measure underlying constructs or psychological dimensions. Therefore, psychometric analyses such as construct validity, factor analysis, or internal consistency reliability (e.g., Cronbach’s alpha) were not applicable and were not performed. This approach aligns with recommendations for the development of short descriptive survey tools in cross-sectional designs where no latent constructs are intended to be measured.

### 2.4 Data collection

Data were collected through an anonymous online questionnaire administered via Google Forms®. Parents and/or legal guardians who attended the Paediatric Dentistry Master's Clinic at Universidad Alfonso X el Sabio were invited to participate consecutively during a 5-month period, from July to November 2024. The Google Forms® survey was not disseminated through social media or external mailing lists to ensure that only eligible participants were invited and reduced the risk of uncontrolled online recruitment or self-selection through social media exposure. Instead, a secure link or QR code was provided in person to parents or legal guardians attending the paediatric dentistry clinic during their appointments. Participation was voluntary, and informed consent was embedded at the start of the digital form. Once submitted, all responses were automatically stored in the secure Google Workspace environment associated with the university account, accessible only to the research team. No identifiable personal data were collected. Data were downloaded in encrypted format and stored on password protected institutional computers for analysis.

### 2.5 Statistical analysis

A descriptive analysis of the sample was conducted using counts and percentages. Inferential analysis was performed to assess the impact of variables such as sex, educational level, family income, and age of the respondents on aspects related to internet searches, including reasons for searching, search frequency, types of electronic devices used, initial search options, information verification, and the perceived reliability or truthfulness of the information. Bivariate analyses were conducted to examine the association between sociodemographic variables (gender, age, educational level, and family income) and multiple aspects of online information-seeking behaviour. Specifically, each sociodemographic variable was paired with: whether respondents searched for OH information online; the reason for searching; the frequency of consultations; the electronic device used; the first option consulted; the method of information verification; and the perceived reliability of the information. Depending on the distribution of the data and expected frequencies, Chi-square (*χ*^2^) tests or Fisher’s exact tests were applied. *χ*^2^ test was applied when expected frequencies met the required assumptions; otherwise, Fisher’s exact test was used. Statistical analysis was conducted with IBM SPSS Statistics software (version 24, Armonk, NY, USA), with a confidence level of 95% (*p* < 0.05) and asymptotic or bilateral significance.

### 2.6 Ethics approval and consent to participate

The study protocol received ethical approval from the Research Ethics Committee of Universidad Alfonso X el Sabio (Resolution 2024_4/266). All procedures were carried out in accordance with the Declaration of Helsinki and institutional guidelines. Participation was voluntary, and informed consent was obtained digitally from all parents and/or legal guardians prior to completing the questionnaire.

## 3 Results

### 3.1 Sociodemographic characteristics of respondents

A total of 112 surveys were collected. About demographic data, significant asymmetry was observed in the gender distribution of the respondents (*p* < 0.001), with 70.5% identifying as female. The distribution by age, educational level, and income level was also non-uniform (*p* < 0.001), with a higher percentage of respondents falling within the 31–40 age range, holding university or higher vocational qualifications, and reporting total monthly family incomes between €2,000 and €4,000 (Table 1).

**Table 1:** Background profile of the study participants.

Item	Response options	N (%)	95% Confidence Interval		ꭓ^2^ *p* value
			Lower	Upper	
Gender	Male	33 (29.5%)	24	43	<0.001*
	Female	79 (70.5%)	69	88	
Age	<30	22 (19.6%)	15	31	<0.001*
	31–40	64 (57.1%)	54	74	
	>41	26 (23.2%)	18	35	
Educational Level	Primary Education (Elementary School Graduate)	2 (1.8%)	0	6	<0.001*
	Secondary Education (Compulsory Secondary Education, High School Diploma, Intermediate Vocational Training)	22 (19.6%)	15	31	
	Advanced Vocational Training	42 (37.5%)	32	52	
	University Education (Bachelor's Degree, Master's Degree, Doctorate)	46 (41.1%)	36	56	
Familiar Income	<€2,000	26 (23.2%)	18	35	<0.001*
	€2,000–4,000	59 (52.7%)	49	69	
	€4,000–6,000	18 (16.1%)	11	27	
	>€6,000	9 (8%)	5	16	

*Statistically significative. *p* value < 0.05.

### 3.2 Use of social media and digital platforms

The web pages familiar to the respondents, and search habits were analysed (Table 2). The most prevalent was Facebook® with 83.9% of the total, followed by YouTube® (83%), Instagram® (73.2%), Wikipedia® (51.8%), Twitter® (40.2%), maternity-related websites (35.7%), private websites of medical centres, hospitals, or dental clinics (27.7%), biomedical libraries or databases (26.8%), personal blogs (22.3%), and online discussion forums (16.1%). Regarding the type of information sought on social media or digital platforms, 65.2% use the internet to search for current news, followed by health and wellness topics (52.7%), diet and recipes (40.2%), fashion and beauty (37.5%), or sports (25.9%).

**Table 2:** Frequency and percentage of response options among the study sample, with significance values.

Item	Response options	N (%)	95% confidence interval		ꭓ^2^
			Lower	Upper	*p* value
Do you often look for information online about your child’s OH or dental treatments?	No	43 (38.7%)	33	53	<0.001*
	Yes	68 (61.3%)	58	78	
	TOTAL	111 (100%)	-	-	
Why do you search for dental treatment information online?	Interest in the topic	49 (59.8%)	40	57	<0.001*
	Did not understand the explanation	2 (2.4%)	0	6	
	To verify professional information	7 (8.5%)	3	13	
	Insufficient information provided	5 (6.1%)	1	8	
	Distrust of the diagnosis	1 (1.2%)	2	11	
	Combination	15 (18.3%)	0	5	
	No sources	3 (3.7%)	9	23	
	TOTAL	80 (100%)	-	-	
Which electronic devices do you use to look for information?	Mobile phone	55 (60.4%)	46	64	<0.001*
	Tablet	1 (1.1%)	0	5	
	Personal Computer	1 (1.1%)	0	5	
	Personal Computer and mobile phone	20 (22%)	13	28	
	Personal Computer, tablet and mobile phone	6 (6.6%)	3	12	
	Tablet and mobile phone	7 (7.7%)	3	13	
	Personal Computer and tablet	1 (1.1%)	0	5	
	TOTAL	91 (100%)	-	-	
Which source do you consult first?	Maternity web pages	24 (26.7%)	17	33	<0.001*
	Scientific literature databases	15 (16.7%)	9	23	
	Google	6 (6.7%)	3	12	
	Instagram®	19 (21.1%)	12	27	
	Parenting forums	5 (5.6%)	2	11	
	Twitter	1 (1.1%)	0	5	
	Wikipedia	8 (8.9%)	4	14	
	Facebook®	11 (12.2%)	6	18	
	YouTube®	1 (1.1%)	0	5	
	TOTAL	90 (100%)	-	-	
Do you verify the information you find with anyone?	Family or friend	32 (34.8%)	24	41	<0.001*
	General dentist	25 (27.2%)	17	34	
	Paediatric Doctor	13 (14.1%)	8	21	
	Paediatric Dentist	20 (21.74%)	13	28	
	No	2 (2.2%)	0	6	
	TOTAL	92 (100%)	-	-	
How reliable do you consider online information?	Unreliable	5 (4.5%)	2	11	<0.001*
	Not very reliable	54 (48.2%)	44	64	
	Moderately reliable	47 (42%)	37	57	
	Very reliable	6 (5.4%)	3	12	
	TOTAL	112 (100%)	-	-	
How often do you search online for your child’s oral health?	Never	30 (27.5%)	22	40	0.120
	At least once a month	28 (25.7%)	20	38	
	At least once a year	34 (31.2%)	25	44	
	Less than once a year	17 (15.6%)	11	25	
	TOTAL	109 (100%)	-	-	

*Statistically significative. *p* value < 0.05.

### 3.3 Use of social media to obtain general and OH information

The majority (61.3%) of participants reported that they search for information about their children's OH and dental treatment on social media or digital platforms. Of these, 59.8% indicated that their searches were motivated by a personal interest in the topic, which was significantly more common than other reasons (*p* < 0.001). The mobile phone was the most often used device for information searches (60.4%) (*p* < 0.001), followed by a combination of the mobile phone and personal computer (22%) (*p* < 0.001). Analysis of the primary search options revealed that maternity websites (26.7%), Instagram® (21.1%), and scientific databases (16.7%) were the most frequently used, surpassing other sources (*p* < 0.001). For verification methods, 34.8% of participants reported consulting with family and friends, while 27.2% sought advice from a general dentist and 21.74% from a paediatric dentist (*p* < 0.001). It is notable that 52.7% of respondents rated the reliability of the information sources they consulted as either not very reliable or not reliable at all.

Respondents were asked to identify which digital platforms or social media they perceived as the most reliable sources for obtaining OH information. The Ministry of Health website and dental clinic websites were considered the most trustworthy (by 39.3% and 36.6% of respondents, respectively), followed by Instagram® (25.9%), the WHO website (23.2%), Facebook® (21.4%), and YouTube® (11.6%). Online discussion forums were considered reliable by 10.7% of participants, Medline Plus by 9.8%, and various parenting websites by 4.5%–8%. Personal blogs were considered reliable by 3.6% of respondents, Google by 1.8%, and Twitter by 0.9%.

**Figure 1:**
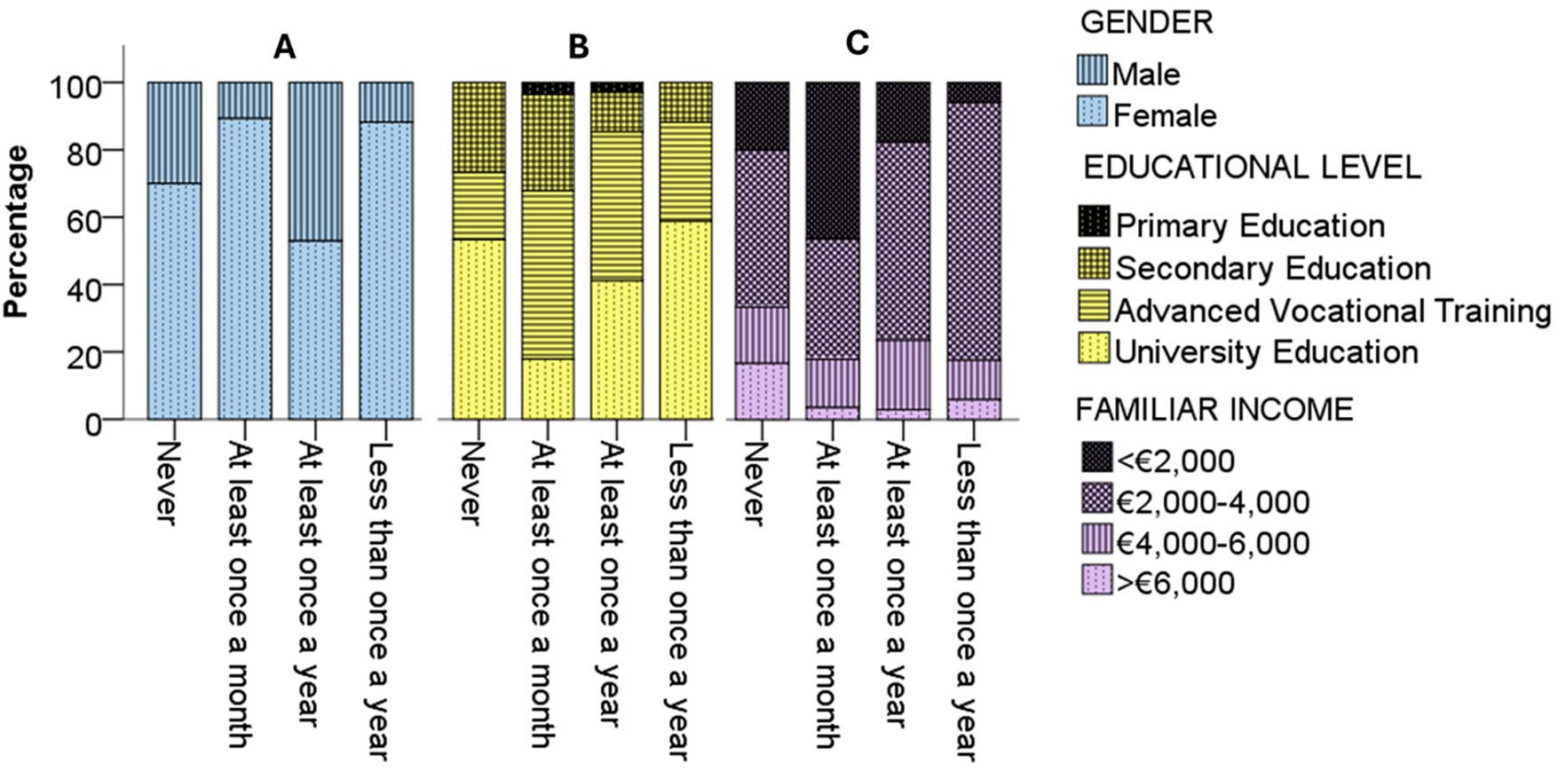
Bar chart of the frequency habits of parental sources according to gender **(A)**, educational level **(B)** and familiar income **(C)**.

In the bivariate analysis (Table 3), no significant differences (*p* > 0.05) were observed about gender, level of academic education, and family income concerning internet searches, reasons for searching, electronic devices used, the first consulted choice, verification methods, or the reliability attributed to the information received. However, the results suggests that information-seeking is more frequent among females, parents with intermediate educational attainment (especially Advanced Vocational Training), and those with lower family income (<€2,000), who show a larger share in the ‘at least once a month’ category, whereas males, university-educated, and higher-income groups are comparatively concentrated in less frequent categories (Figure 1). Specifically, women and families with lower levels of academic education consulted more often (*p* = 0.005 and *p* = 0.033, respectively), and respondents with incomes below €2,000 consulted more frequently compared to those with higher incomes (*p* = 0.044). Interestingly, while most women answered consulting at least once a month, males stated searching for information at least once a year. A relevant result indicated that parents with University Education consulted significantly less a month than lower educational level families, while parents with Advanced Vocational Training rarely responded that “never” searched for information. Besides, although higher familiar income level (>€6,000) was associated to less sources frequency, and incomes between €2,000 and €4,000 searched for information less than once a year, very lower familiar incomes (<€2,000) were associated with higher sources frequency (at least once a month). No significant differences were noted based on the respondent's age (*p* > 0.05).

**Table 3:** Results of statistical tests for intragroup analysis.

Paired comparisons	Sex	Age	Educational level	Income
	*P* value	*P* value	*P* value	*P* value
Internet Sources	0.927^a^	0.904^a^	0.118^b^	0.273^a^
Sources Reasons	0.531^b^	0.140^b^	0.107^b^	0.902^b^
Sources Frequency	0.005^a^^,^*	0.465^a^	0.033^b^^,^*	0.044^b^^,^*
Electronic devices	0.272^b^	0.138^b^	0.543^b^	0.132^b^
First Option	0.399^b^	0.578^b^	0.498^b^	0.854^b^
Verification	0.993^b^	0.671^b^	0.259b	0.059^b^
Confidence	0.244^b^	0.165^b^	0.615^b^	0.318^b^

^a^ Bilateral signification of ꭓ^2^ test.

^b^ Exact signification of Fisher test.

*Statistically significative. *p* value < 0.05.

## 4 Discussion

The present study aimed to analyse how parents seek information on paediatric OH through digital platforms and social media, and to explore their perceptions regarding reliability and verification practices. Overall, the findings suggest that digital media, particularly smartphone-based access, play a central role in parental information-seeking behaviours. Although official health websites and dental professionals were perceived as the most trustworthy sources, parents more frequently consulted maternity websites and social media platforms. These results indicate that accessibility and habitual digital use may outweigh perceived credibility when selecting information sources.

The use of digital platforms and social media as sources of information on various issues and topics of interest in our daily lives is becoming increasingly prevalent. Notably, pregnant women and first-time mothers frequently utilize platforms such as Facebook® to gather information on the care and health of their newborns (10). To analyse the use of digital media for seeking information on Paediatric Dentistry by parents, an online survey was conducted with 112 participants. Over 70% of the respondents were women aged between 30 and 40 years, with university education and monthly incomes ranging from €2,000 to €4,000. The survey revealed that 68% of these participants sought information on social media using mobile phones. These results are consistent with findings from studies on internet information-seeking habits in adults (15). Cross-country comparisons should be interpreted with caution, as socio-cultural differences and levels of digital health literacy may substantially influence parents’ online habits and their appraisal of information; broader evidence shows that higher eHealth literacy is associated with more critical consumption of online OH content and reduced susceptibility to influencer-driven products (16). In parenting contexts, studies during and after the pandemic similarly report persistent, high-frequency smartphone use for caregiving information and underscore opportunities to embed digital-literacy guidance in routine maternal/child care (26). The average parental age (30–40 years), gender (predominantly female), and elevated level of education (university) among respondents align with data reported in other studies (15, 27). However, we found differences in studies conducted in the Middle East. In a study conducted in Saudi Arabia (28), the parental age was younger (20–30 years), but the levels of education and economic status were similar to those in our study. Conversely, in a study conducted in Bahrain (27), the predominant level of education was secondary education. Other authors have also used social media for conducting OH questionnaires regarding children and dental health. Almalki et al. (28) utilized WhatsApp® and Snapchat® for their study on parental attitudes and awareness regarding preventive dentistry, and Nassar et al. (29) employed social media for conducting a questionnaire on parents’ knowledge of early childhood caries (ECC). Abbasi et al. (30) distributed a survey via WhatsApp® and email among dentists to evaluate the impact of aesthetic dentistry among social media users. Nearly half of the surveyed dentists believed that sharing their work on social media is an effective way to promote and disseminate their work.

Related to the main results, Sivaramakrishnan et al. (27) stated that 83.5% of adults used social media to view content related to dentistry, which is a higher percentage compared to the 68% observed in the present study. The use of social media was associated with both viewing dentistry-related content and direct consultations with dentists through these platforms. Therefore, we hypothesize that individuals are likely to conduct more internet and social media searches for themselves than for their children. Consistent with the obtained results, most respondents reported using their personal mobile phones as the primary device for obtaining information from the internet, aligning with the results reported by Bryan et al. (15).

No significant differences were found in the bivariate analysis regarding gender and information seeking through social media, which aligns with the findings of Istl et al. (31), finding no differences between genders in the use of the social media platform Twitter®. However, they noted that most content creators or influencers in the field of medicine are men. In our study, it was observed that the platform most often chosen for information searches was maternity websites, followed by Instagram®. This contrasts with several other studies, such as those by Rivera et al. (32), which identified Facebook® as the primary social network used who only analysed healthcare information from the Facebook® social network. Bryan et al. (15) conducted an analysis on parents’ perceptions regarding the use of the internet and social media to obtain information about paediatric health, with Facebook® being the most utilized platform. However, other social networks have been found related to health search. Sivaramakrishnan et al. (27), after conducting a survey among both dentists and non-healthcare personnel, it was noted that WhatsApp® was more commonly used for exchanging opinions about the information found, with more than 6 h of usage per day, followed by Instagram® and Twitter®. Given that maternity websites and Instagram® were among the most frequently used platforms in our sample, dental professionals and scientific organisations should prioritise these channels to deliver evidence-based, visually accessible content. Social-media interventions can improve multiple OH outcomes, as stated in a systematic review, supporting platform-specific education and behaviour change (33). Moreover, analyses of parenting Instagram® accounts highlight both the reach and the under-utilisation of maternal-health content, indicating a missed opportunity for targeted collaborations with high-traffic pages (34).

Social media can be considered a valid platform for disseminating health messages, due to its widespread accessibility. These platforms can play a significant role in educating parents and caregivers, as well as in promoting preventive behaviours related to oral hygiene, dental check-ups, and the prevention of early childhood caries. In 2017, Gough et al. (35) conducted an analysis on the feasibility of utilizing social media platforms, for disseminating promotional techniques and evaluation measures. They observed that social media platforms, specifically Twitter®, are indeed effective for spreading public health campaigns, given their ability to deliver dynamic and personalized messages to audiences in real-time. However, the authors also emphasized the need for cautious interpretation of survey results obtained via the internet, as these results may not accurately represent the general population’s understanding of public health issues. The use of social media and online platforms should, nowadays, be considered a complementary method to traditional OH education sessions. In fact, it can be affirmed that online social media interventions can contribute to improvements in OH status (36). Modern technologies can aid in the management of OH. For instance, methods such as text messaging have proven effective in controlling dental caries (37, 38), with effectiveness similar to conventional OH promotion appointments.

Regarding the reliability of the information, half of respondents found the information they obtained from social media about paediatric dental health to be somewhat or not reliable. To verify this information, mostly consulted a general dentist or paediatric dentist, and less respondents checked with a family member. The digital platforms or social media considered most reliable for obtaining OH information were the Ministry of Health's website and dental clinic websites, considered the most trustworthy of respondents, respectively, followed by Instagram®. Korshakova et al. (39) found that the sources varied depending on the type of disease: for common illnesses like food allergies, streptococcal pharyngitis, and strokes, individuals relied more on family, friends, and medical professionals. In contrast, for emerging diseases such as Ebola, the common cold, COVID-19, and Zika, media outlets, government agencies, and social media were preferred. The researchers noted that individuals often trust the sources they use, even when these sources, such as social media, are considered to be of low quality. These patterns are consistent with recent Spanish evidence (40) showing that social networks are a predominant source of health information and that active seekers display higher digital-health literacy. The obtained results show that 72.1% of the participants reported actively using social media to search for health information, and active users scored significantly higher on e-Health literacy.

Given that our study found that most respondents consider sources like the Ministry of Health and dentists/paediatric dentists to be the most reliable, we concur with Dhar et al. (41) and emphasize that national and international dental associations and societies should play a significant role in disseminating information through social media, collaborating with scientific committees to ensure the accuracy of information exchanged in these forums. Indeed, a study evaluating children aged 12–15 in England (18) found that those who sought information about their OH on the Internet or social media experienced a higher incidence of decayed teeth compared to those whose primary source of information was their parents. Therefore, we concur with other authors that it is essential to critically evaluate social media content and ensure that it reliably addresses the concerns of users seeking health information on these platforms (25, 42, 43). Thus, Aguirre et al. (44), who analysed 120 YouTube® videos with content related to the aetiology and prevention of ECC, noted that these videos focused on partial aspects of the disease. For this reason, educational videos of higher quality are necessary, highlighting the importance of dental professionals guiding and serving as reliable sources of information. On the other hand, Marocolo et al. (45) analysed the quality of health-related posts on Instagram® and noted that content creators on this social media platform disseminated low-quality information regarding exercise and health, contributing to the widespread dissemination of misinformation among millions of followers. As Swire-Thompson and Lazer (46) affirm, we lack the cognitive capacity, motivation, or time to evaluate all the information available on the internet. However, motivation increases when we research a topic related to our own health conditions or symptoms; even in these circumstances, assessing the reputation of sources and the accuracy of information is an extremely challenging task. Moreover, the internet is a constantly changing system, which makes studying health misinformation even more complex. An interesting finding of this study is the discrepancy between the most trusted sources of OH information and the most frequently used sources, namely maternity websites and Instagram®. This pattern suggests that parental information seeking behaviour is influenced not only by perceived reliability but also by accessibility, familiarity, and the ease of obtaining quick, relatable content. Social media platforms and maternity websites offer a more informal, user-friendly environment and are often integrated into parents’ daily digital routines, which may encourage frequent use even when the information is not considered highly reliable. Additionally, algorithms and peer generated content may increase exposure to these platforms, reinforcing habitual use. A notable gap was observed between parents’ frequent use of online platforms to search for information and their low rate of professional verification. Although more than half of respondents considered the information found online to be ‘not very reliable’ or ‘not reliable at all’, only 21.74% sought confirmation from a paediatric dentist. This discrepancy highlights a critical disconnect between awareness of unreliability and engagement with qualified professionals. Several factors may contribute to this pattern, including convenience, limited time, the immediacy of online information, and reliance on informal networks such as family and friends. It also suggests that parents may not always perceive OH concerns as urgent enough to justify contacting a dentist, or that they may lack easy access to professional guidance outside scheduled appointments.

It's important to consider that social media is also used to disseminate new complementary medicines or alternative therapies to users. In this regard, Gülpinar et al. (47), in 2023, concluded on the importance of regulating these practices and analysing the impact of social media influencers on these topics. As we have mentioned before, the information may not be entirely reliable, and users of such treatments or therapies may not achieve the results advertised on the internet. Furthermore, it's a fact that the percentage of digital natives is increasing, and soon, Generation Z (born from the 1990s and early 2000s) will join dentistry as professionals. The prominent role of influencers in shaping parental perceptions necessitates a proactive stance from dental professionals, and collaboration with credible influencers to product of verified, platform-specific content to enhance quality and reach (48). From a public-health perspective, tackling health misinformation calls for multi-layered strategies (communication, technology-based and multimedia approaches) and active participation of reputable health organisations and influencers to increase effectiveness (49). Complementarily, the broader public-health literature frames misinformation on social media as a threat requiring coordinated primary, secondary, and tertiary preventive actions, including debunking, warning labels, literacy “nudges,” and system-level regulation (50).

Regarding the limitations of this study, its cross-sectional nature and reliance on self-reported surveys completed by parents or legal guardians are notable. Self-administered questionnaires are prone to recall and social-desirability bias, and common-method variance cannot be excluded. Additionally, the rapid evolution and modification of internet products, social media, and digital applications complicate comparisons between them, as they are subject to generational, economic, and personal factors. The use of a non-probabilistic consecutive sampling method constitutes an important limitation of the present study. Because all participants were recruited from a single university-based paediatric dentistry clinic, the sample may differ from the broader population of parents in terms of education level, socioeconomic status or health-seeking behaviour. Parents who attend a university clinic may be more engaged in preventive care or may have greater digital literacy, which could influence their patterns of online information seeking. Consequently, the findings should be interpreted with caution, and generalizability to the wider population may be limited. Although the required minimum sample size was calculated as 103 participants, the final sample consisted of 112 respondents. Exceeding the calculated sample does not compromise statistical power; instead, it marginally increases the precision of the estimates. However, representativeness is still limited by the non-probabilistic sampling strategy and the single-centre recruitment.

An additional limitation relates to the sociodemographic composition of the sample. The majority of participants were women aged 31–40 with university level education. This profile may not reflect the wider parent population, as mothers are generally more involved in childcare related decision-making and tend to seek online health information more actively than fathers. The predominance of female participants reflects the common pattern in paediatric healthcare visits, where mothers attend more frequently than fathers. However, this limits the representativeness of the sample and may reduce generalizability to the broader parent population. Therefore, findings should be interpreted within the context of this demographic imbalance. Higher educational attainment may also be associated with greater digital literacy and health-seeking behaviour, potentially influencing the patterns observed. Therefore, the findings should be interpreted with caution, as the overrepresentation of this subgroup may limit generalizability. Furthermore, multiple bivariate comparisons increase the risk of type-I error; although Fisher's exact tests were used when expected counts were small, future analyses could reduce multiplicity via parsimonious modelling and apply post-stratification/weighting to better approximate target populations.

Regarding instrument development, content validity was assessed by a small expert panel (*n* = 3) and face/usability testing was conducted in a limited pilot (*n* = 12). These numbers are acceptable for preliminary clarity checks but reduce the robustness of validation and should be interpreted cautiously. Because the questionnaire comprised exclusively descriptive, categorical, non-latent items, psychometric procedures intended for reflective latent constructs were not applicable; this follows established measurement guidance distinguishing formative/descriptive from reflective indicators, for which internal consistency and factor modelling are neither required nor informative. Our multi-step process is consistent with international recommendations for evaluating content validity and clarity.

A strength of this study is its timeliness, as current research is focusing on tele-dentistry models to complement in-person appointments. Tele-dentistry and Internet of Things (IoT)-based healthcare services (51, 52) currently offer numerous advantages, such as remote consultations, real-time monitoring, and gamification applications, which can facilitate access to dental care for patients facing geographical barriers, dental anxiety, or phobia, or those who are institutionalized or hospitalized.

Despite the influence that innovative technologies may have on dentistry, the vast majority of the population still states that the primary reason for choosing a dentist remains the recommendation of a friend or family member (93.5%) or the professional’s curriculum (47.1%). However, factors such as content posted on social media (23.6%), the number of social media followers (7.1%), and endorsements from influencers (6%) are gaining traction (16). Therefore, it is crucial for paediatric dentists to understand the digital habits of parents and/or caregivers to foster a more personalized approach and build closer relationships.

The findings of this study highlight several opportunities for strengthening OH promotion in the digital environment. Public health practitioners should prioritize the development of accessible, evidence-based online resources aimed at parents and caregivers, ensuring that reliable information is available on the platforms they most frequently use. Dental professionals and scientific organizations are encouraged to collaborate with digital platforms and content creators to enhance the visibility and credibility of accurate OH messages. Policymakers should support initiatives that improve eHealth literacy, particularly among younger parents, by integrating training on how to critically evaluate online health information into community and maternal-child health programs. Additionally, regulatory frameworks should be strengthened to promote transparency, protect user privacy, and limit the dissemination of misinformation related to children's OH. Future interventions should be designed to combine digital communication with traditional clinical guidance, ensuring that families receive consistent, trustworthy recommendations across all channels. Collaborations between OH professionals, organizations, and social media content creators can enhance both credibility and outreach. However, such advances also raise ethical considerations, requiring strict attention to privacy regulations and proactive efforts to counter misinformation. Strengthening parents’ digital health literacy is likely to reduce susceptibility to misinformation and improve appraisal of online content., as higher eHealth literacy has been associated with more critical consumption of OH (16). Programs embedded in maternal-and-child health that integrate app-supported information-seeking have shown associations with better parental knowledge and literacy scores, underscoring a practical route for clinical and public-health services to amplify the impact of professional content on social platforms (53).

## 5 Conclusions

The findings of this study show that while a majority of parents (61.3%) search for information on their children’s OH through online platforms, particularly maternity websites, scientific databases, and Instagram®, only 21.74% verify this information with a paediatric dentist. This discrepancy suggests that accessibility and familiarity strongly shape parental information-seeking behaviour, even though many respondents consider online content only moderately reliable. These patterns underscore a growing need for OH professionals to strengthen their visibility within the digital spaces that parents already use.

Looking ahead, social media should serve as a complementary channel for OH promotion, enhanced through the creation of verified, engaging, and platform-specific content. Dental professionals and public-health organisations have an opportunity to counter misinformation by collaborating with credible creators, integrating digital health-literacy strategies into maternal and child-health programmes, and leveraging emerging tools such as tele-dentistry and personalised digital communication. Adapting to these evolving digital environments will be essential for ensuring that parents receive accurate, accessible, and trustworthy OH guidance.

## Data Availability

Due to restrictions imposed by the Research Ethics Committee of Universidad Alfonso X el Sabio (Resolution 2024_4/266) and General Data Protection Regulation obligations, the raw individual-level data cannot be publicly shared because of the non-negligible risk of indirect re-identification in this small sample and the absence of participant consent for data redistribution. Aggregated data supporting the findings are included in the article, and additional derived outputs may be provided upon reasonable request to the corresponding authors. Requests to access the datasets should be directed to areyeort@uax.es; amartvac@uax.es.
