# Supplementary material for: Use of digital platforms and social media as a source of information on children's oral health by parents: a cross-sectional survey analysis in a Spanish sample
Source: Front Oral Health. 2026 Mar 24;7:1754009. doi: 10.3389/froh.2026.1754009 (PMC13055506; doi:10.3389/froh.2026.1754009)
Supplement: Supplementary file 1 [file Table1.docx]

**Appendix 1.** STROBE checklist (21) of items included in the manuscript.

|  | | Item No | | | Recommendation | Location in the manuscript | |
| --- | --- | --- | --- | --- | --- | --- | --- |
| Title and abstract | | | | | | | |
|  | | 1 | | | (*a*) Indicate the study’s design with a commonly used term in the title or the abstract | Title; Abstract | |
|  |  |  |  |  | (*b*) Provide in the abstract an informative and balanced summary of what was done and what was found | Abstract | |
| Introduction | | | | | |  | |
| Background/rationale | | 2 | | | Explain the scientific background and rationale for the investigation being reported | Introduction | |
| Objectives | | 3 | | | State specific objectives, including any prespecified hypotheses | Introduction | |
| Methods | | | | | |  | |
| Study design | | 4 | | | Present key elements of study design early in the paper | Methods - Study design | |
| Setting | | 5 | | | Describe the setting, locations, and relevant dates, including periods of recruitment, exposure, follow-up, and data collection | Methods - Data collection | |
| Participants | | 6 | | | (*a*) *Cross-sectional study*—Give the eligibility criteria, and the sources and methods of selection of participants | Methods - Study population | |
|  |  |  |  |  | (*b*) *Cohort study*—For matched studies, give matching criteria and number of exposed and unexposed  *Case-control study*—For matched studies, give matching criteria and the number of controls per case | No applicable | |
| Variables | | 7 | | | Clearly define all outcomes, exposures, predictors, potential confounders, and effect modifiers. Give diagnostic criteria, if applicable | Methods - Questionnaire description | |
| Data sources/ measurement | | 8* | | | For each variable of interest, give sources of data and details of methods of assessment (measurement). Describe comparability of assessment methods if there is more than one group | Methods - Study design | |
| Bias | | 9 | | | Describe any efforts to address potential sources of bias | Methods; Discussion - Limitations | |
| Study size | | 10 | | | Explain how the study size was arrived at | Methods - Sample size | |
| Quantitative variables | | 11 | | | Explain how quantitative variables were handled in the analyses. If applicable, describe which groupings were chosen and why | Methods - Statistical analysis | |
| Statistical methods | | 12 | | | (*a*) Describe all statistical methods, including those used to control for confounding | Methods - Statistical analysis | |
|  |  |  |  |  | (*b*) Describe any methods used to examine subgroups and interactions | Methods - Statistical analysis | |
|  |  |  |  |  | (*c*) Explain how missing data were addressed | Methods - Statistical analysis | |
|  |  |  |  |  | (*d*) *Cross-sectional study*—If applicable, describe analytical methods taking account of sampling strategy | Methods – Study population and sample size calculation | |
|  |  |  |  |  | (*e*) Describe any sensitivity analyses | Not applicable | |
| Results | | | | | |  | |
| Participants | 13* | | | (a) Report numbers of individuals at each stage of study—eg numbers potentially eligible, examined for eligibility, confirmed eligible, included in the study, completing follow-up, and analysed | | Results - Sociodemographic characteristics | |
|  |  |  |  | (b) Give reasons for non-participation at each stage | | Not applicable | |
|  |  |  |  | (c) Consider use of a flow diagram | | Not applicable | |
| Descriptive data | 14* | | (a) Give characteristics of study participants (eg demographic, clinical, social) and information on exposures and potential confounders | | | | Results; Tables 1-2 |
|  |  |  | (b) Indicate number of participants with missing data for each variable of interest | | | | Results; Tables 1-2 |
|  |  |  | (c) *Cohort study*—Summarise follow-up time (eg, average and total amount) | | | | Not applicable |
| Outcome data | 15* | | *Cross-sectional study—*Report numbers of outcome events or summary measures | | | | Results |
| Main results | 16 | | (*a*) Give unadjusted estimates and, if applicable, confounder-adjusted estimates and their precision (eg, 95% confidence interval). Make clear which confounders were adjusted for and why they were included | | | | Results; Table 3 |
|  |  |  | (*b*) Report category boundaries when continuous variables were categorized | | | | Methods |
|  |  |  | (*c*) If relevant, consider translating estimates of relative risk into absolute risk for a meaningful time period | | | | Not applicable |
| Other analyses | 17 | | Report other analyses done—eg analyses of subgroups and interactions, and sensitivity analyses | | | | Results; Figure 1 |
| Discussion | | | | | | | |
| Key results | 18 | | Summarise key results with reference to study objectives | | | | Discussion |
| Limitations | 19 | | Discuss limitations of the study, taking into account sources of potential bias or imprecision. Discuss both direction and magnitude of any potential bias | | | | Discussion - Limitations |
| Interpretation | 20 | | Give a cautious overall interpretation of results considering objectives, limitations, multiplicity of analyses, results from similar studies, and other relevant evidence | | | | Discussion |
| Generalisability | 21 | | Discuss the generalisability (external validity) of the study results | | | | Discussion |
| Other information | | | | | | | |
| Funding | 22 | | Give the source of funding and the role of the funders for the present study and, if applicable, for the original study on which the present article is based | | | | Funding section |

**Appendix 2.** 14-items questionnaire in English with original Spanish in italics.

| **Question** | **Response options** |
| --- | --- |
| **1. Indicate your gender**  *Indique su género* | Male; Female; Other  *Masculino; Femenino; Otro* |
| **2. Which of the following age ranges do you fall into?**  *¿Entre cuál de los siguientes rangos de edad se encuentra?* | Under 30 years; 31–40 years; Over 41 years  *Menor de 30 años; 31–40 años; Más de 41* |
| **3. What is the highest level of education you have completed?**  *¿Cuál es el máximo nivel de estudios que ha completado?* | Primary education (Elementary School Graduate); Secondary education (Compulsory Secondary Education, High School Diploma, Intermediate Vocational Training); Advanced Vocational Training; University education (Bachelor’s degree/Master’s degree/PhD Doctorate)  *Primarios (Graduado Escolar); Secundarios (ESO, Bachillerato, Ciclo Medio); Ciclo Formativo de Grado Superior; Universitarios (Grado/Máster/Doctorado)* |
| **4. What is your household’s total monthly income?**  *¿Cuál es el nivel de ingresos mensuales de su unidad familiar?* | €0–€2,000; €2,001–€4,000; €4,001–€6,000; >€6,000  *0–2.000€; 2.000–4.000€; 4.000–6.000€; >6.000€* |
| **5. Which of the following websites/platforms are you familiar with?**  *¿Con cuál de los siguientes sitios web está familiarizado?* | Wikipedia; Instagram; Facebook; YouTube; Maternity websites; Private hospital/dental clinic websites; Discussion forums; Personal blogs; Twitter/X; Scientific literature databases  *Wikipedia; Instagram; Facebook; YouTube; Páginas de maternidad; Sitios de hospitales/clínicas dentales; Foros de discusión; Blogs personales; Twitter; Bibliotecas/bases de artículos científicos* |
| **6. What type of information do you most often search for on the Internet/social media?**  *¿Qué tipo de información busca en Internet/Redes sociales?* | Fashion/Beauty; Sports; Diet/Recipes; Health & Wellness; Current news; Other  *Moda/Belleza; Deporte; Dieta/Recetas; Salud y Bienestar; Noticias de actualidad; Otra* |
| **7. Do you often look for information online about your child’s oral health or dental treatments?**  *¿Busca habitualmente información en Internet sobre la salud oral/tratamientos dentales de su hij@?* | Yes; No  *Sí; No* |
| **8. Why do you search for dental treatment information online?**  *¿Por qué busca información sobre tratamientos dentales en Internet?* | Interest in the topic; To verify professional information; Distrust of the diagnosis; Did not understand the explanation; Insufficient information provided; Combination; No sources  *Interés en el tema; Contrastar información del profesional; Desconfianza hacia el diagnóstico; No entendí la explicación; No me dieron suficiente información; Combinación; No búsquedas* |
| **9. How often do you search online for your child’s oral health?**  *¿Con qué frecuencia utiliza Internet/sitios web para buscar información sobre la salud oral de su hij@?* | At least once a month; At least once a year; Less than once a year; Never  *Al menos una vez al mes; Al menos una vez al año; Menos de una vez al año; Nunca* |
| **10. Which electronic devices do you use to look for information?**  *¿Qué dispositivos electrónicos utiliza para buscar información?* | Personal Computer; mobile phone; Tablet; Other  *Ordenador personal; teléfono móvil; Tablet; Otro* |
| **11. Which source do you consult first?**  *¿A cuál de las siguientes opciones recurre primero?* | Scientific literature databases; Wikipedia; Google; Maternity websites; Instagram; Twitter; Facebook; YouTube; Parenting forums; Other  *Bibliotecas/bases de artículos científicos; Wikipedia; Google; Páginas de maternidad; Instagram; Twitter; Facebook;YouTube; Foros de padres; Otra* |
| **12. Do you verify the information you find with anyone?**  *¿Hay alguien con quien verifique la información que encuentra en Internet?* | Family or Friends; General dentist; Paediatric Doctor; Paediatric dentist; No; Other  *Familiar o amigo; Odontólogo general; Pediatra; Odontopediatra; No; Otra* |
| **13. Which of the following do you consider reliable sources?**  *¿Cuáles considera fuentes fiables?* | Dental clinic websites; Ministry of Health website; TodoPapás; HealthyChildren; WHO website; Instagram; EnFemenino; MedlinePlus; Facebook; Parenting forums; Personal blogs; Crianza Natural; YouTube; Other  *Páginas de clínicas dentales; Ministerio de Sanidad; TodoPapás; HealthyChildren; Página de la OMS; Instagram; EnFemenino; MedlinePlus; Facebook; Foros de padres; Blogs personales; Crianza Natural; YouTube; Otra* |
| **14. How reliable do you consider online information?**  *¿Qué grado de confianza le otorga a la información extraída de Internet?* | Unreliable; Not very reliable; Moderately reliable; Very reliable  *Nada fiable; Poco fiable; Suficientemente fiable; Muy fiable* |
